# Resveratrol, 4′ Acetoxy Resveratrol, *R*-equol, Racemic Equol or *S*-equol as Cosmeceuticals to Improve Dermal Health

**DOI:** 10.3390/ijms18061193

**Published:** 2017-06-03

**Authors:** Edwin D. Lephart

**Affiliations:** Department of Physiology and Developmental Biology and The Neuroscience Center, LS 4005, College of Life Sciences, Brigham Young University, Provo, UT 84602, USA; Edwin_Lephart@byu.edu; Tel.: +1-801-422-2006; Fax: +1-801-422-0700

**Keywords:** phytochemicals, resveratrol, equol, human skin, gene array, extracellular matrix proteins, antioxidant, anti-inflammatory

## Abstract

Phytochemicals are botanical compounds used in dermatology applications as cosmeceuticals to improve skin health. Resveratrol and equol are two of the best-known polyphenolic or phytoestrogens having similar chemical structures and some overlapping biological functions to 17β-estradiol. Human skin gene expression was reviewed for 28 different biomarkers when resveratrol, 4′ acetoxy resveratrol (4AR), *R*-equol, racemic equol or *S*-equol were tested. Sirtuin 1 activator (SIRT 1) was stimulated by resveratrol and 4AR only. Resveratrol, *R*-equol and racemic equol were effective on the aging biomarkers proliferating cell nuclear factor (PCNA), nerve growth factor (NGF), 5α-reductase and the calcium binding proteins S100 A8 and A9. Racemic equol and 4AR displayed among the highest levels for the collagens, elastin and tissue inhibitor of the matrix metalloproteinase 1 (TIMP 1). *S*-equol displayed the lowest level of effectiveness compared to the other compounds. The 4AR analog was more effective compared to resveratrol by 1.6-fold. *R*-equol and racemic equol were almost equal in potency displaying greater inhibition vs. resveratrol or its 4′ analog for the matrix metalloproteinases (MMPs), but among the inflammatory biomarkers, resveratrol, 4AR, *R*-equol and racemic equol displayed high inhibition. Thus, these cosmeceuticals display promise to improve dermal health; however, further study is warranted to understand how phytochemicals protect/enhance the skin.

## 1. Introduction

### 1.1. Phytochemicals

Phytochemicals are botanical compounds that have gained popularity due to increased emphasis on complementary and alternative medicine (CAM) and their use in many commercial products. Remarkably, the use of CAM products was reported to be significantly higher in adults with skin disease (49.4%) as compared to the general population (36.0%) [1]. From a dermatology perspective, cosmeceuticals represent the blending of cosmetics and pharmaceuticals [2,3,4]. The term cosmeceutical was first coined by Albert Kligman in 1984 to describe topical products that afford both cosmetic and therapeutic benefits [5]. Botanicals from nature or plant-derived compounds that subsequently have been improved upon in the laboratory make up a large percentage of active ingredients in cosmeceuticals [2,3,4]. Cosmeceuticals (such as sunscreens and moisturizers) may be as effective over time for many individuals and assuredly less expensive compared to surgery.

### 1.2. Steroid Hormones and Estrogen Receptors: Implications for Phytochemicals

However, it is well established that steroid hormones have a significant impact on dermal fitness, where estrogens protect, enhance and improve skin health, whereas androgens oppose estrogen’s positive actions in human skin [6,7,8,9]. Plant-derived polyphenolic compounds have similar ring-like structures to that of 17β-estradiol, as well as comparable molecular weights, and many are classified as phytoestrogens due to their ability to bind human estrogen receptors that suggest analogous mechanisms of action [10,11,12].

### 1.3. Publications of: Polyphenolic or Phytoestrogen Molecules

Based on the number of publications in peer-reviewed journals within the past six years, it is indicated that two of the most well-known polyphenolic/phytoestrogenic molecules are resveratrol, derived from grapes, and equol, an isoflavonoid found in plant and food products, and it is also produced by intestinal bacteria conversion from daidzein [13,14,15,16].

### 1.4. Resveratrol and Its Metabolism

For resveratrol, the dramatic increase in research interest occurred in early 1997, when its chemoprotective effects were first reported in Science by John Pezzuto’s laboratory [13]. This report showed the potential of resveratrol to prevent tumor initiation, promotion and progression that may be used as a potential anti-cancer agent. Nevertheless, resveratrol suffers from rapid phase II metabolism where almost all of the compound is conjugated when taken orally, resulting in very low bioavailability [15].

### 1.5. Analogs of Resveratrol

In this regard, in order to address this potential challenge, our laboratory along with others synthesized resveratrol analogs to enhance physical/chemical properties to improve the effectiveness in dermal applications. For example, Ryu et al. synthesized the resveratrol analog, resveratryl triacetate [17]. Their report suggested that this resveratrol analog, with increased stability, may be incorporated into cosmetic formulations that can whiten human skin without skin irritation [17]. In this effort, our laboratory developed the novel synthesis of a resveratrol analog (i.e., 4′ acetoxy-resveratrol) to obtain enhanced stability and bioavailability for cosmetic utility in order to advance dermal health, along with other applications [18]. In this manner, natural resveratrol could then be compared to our new 4′ acetoxy resveratrol analog in gene array studies, since natural resveratrol appears to be an effective dermal treatment in antioxidant skin care formulations [19,20,21].

### 1.6. Equol

While not as dramatic as resveratrol in research attention, in the late 1990s, the “equol hypothesis” was proposed, which suggested that maintaining blood levels of equol at threshold concentrations (around 10 to 20 ng/mL) would imply health benefits in humans, such as protection against breast and prostate cancer [16]. After the “equol hypothesis” was proposed, a rapid increase in research activity occurred that examined its beneficial properties for a variety of disorders [16].

### 1.7. Isomers of Equol

For example, equol has a chiral center at carbon 3 and, thus, can exist in two mirror image forms known as enantiomers (*S*-equol and *R*-equol) [14,16]. Racemic equol refers to exact equal portions of *S*-equol and *R*-equol that can be biosynthesized from daidzein at high purity (≥99%) [14]. Remarkably, in 2008, some scientific reports suggested that not all enantiomers or isomers in organic systems are biologically active [22]. For example, although many biologically-active molecules are chiral, including the naturally occurring amino acids and some sugars, most of these compounds are of the same chirality [22]. Therefore, some scientific perspectives displayed a belief that it would not be expected for both enantiomers and isomers to possess biological functions in vivo. Conversely, the seminal report by Strong, in 1999, stated that “too often, and even without it being noticed, data in the scientific literature on mixtures of stereoisomers, racemates, are presented as if only one compound were involved” [23]. Thus, through investigational studies, we now know that *R*-equol and *S*-equol isomers are unique compounds with specific biological actions that also, in some cases, have overlapping functions that could not be predicted by prior scientific knowledge [14].

### 1.8. Equol in Plant and Food Products

Additionally, *S*-equol has also been found in plant products such as beans, cabbage, lettuces, tofu and other food and animal products like eggs and cow’s milk, as reviewed elsewhere [14]. Notably, the metabolism of *R*- and *S*-equol in humans appears to be similar [16]. Therefore, humans are exposed to these types of polyphenolic and isoflavonoid compounds from different plant and food sources regardless of age, gender or geographical location with scientific data to support a consumption/exposure record that appears to be safe [14].

### 1.9. Resveratrol and Equol Conjugation

The elucidation of the metabolic pathway of equol has been studied in rat, monkey and man where it is also conjugated to a high degree by phase II metabolism, when taken orally, but is more bioavailable compared to orally-administered resveratrol [15,24].

### 1.10. Resveratrol and Equol Applications for Dermal Health

Both resveratrol and equol have been proposed as viable approaches for treating aging skin in humans due to their multi-mechanistic properties [14,19,20,21], and polyphenolic compounds have been proposed for use in dermatologic oncology [25].

### 1.11. Review Topics Covered in this Overview

Therefore, this review summarizes the available data that characterize the chemical properties and gene array results for human skin-related biomarkers when five cosmeceutical compounds were tested: resveratrol, 4′ acetoxy resveratrol, *R*-equol, racemic equol and *S*-equol, under similar laboratory conditions.

Specifically, in brief, this overview covers the following main topics: First, the classification of the five polyphenolic/phytoestrogenic compounds as cosmeceuticals, namely resveratrol, 4′ acetoxy resveratrol, equol and its isomers (*R*-equol and *S*-equol), was compared to 17β-estradiol for chemical structural characteristics and attributes that are presented in Section 2. Next, the estrogen receptor (ER) binding features for the five cosmeceuticals resveratrol, 4′ acetoxy resveratrol, racemic equol, *R*-equol and *S*-equol were compared to 17β-estradiol as a reference standard, which are presented in Section 3 (using quantitative or relative binding affinity data). Finally, in Section 4.1, a general introduction to gene array studies is given, and the topic of cosmeceuticals in Section 4.2 is presented, followed by Section 4.3, which covers phase II metabolism in skin. Then, Section 4.4, the main section on the human gene expression of various dermal-related biomarkers that were quantified for resveratrol, 4′ acetoxy resveratrol, racemic equol, *R*-equol or *S*-equol, is presented so that comparisons and rankings could be made among the cosmeceuticals tested under similar laboratory conditions. In the subsequent subsections, for the skin biomarkers, the main topics include: anti-aging gene expression in Section 4.4.2, extracellular matrix proteins in Section 4.4.3, inhibitors of matrix metallo-proteinases (MMPs) or, in other words, tissue inhibitor of the matrix metallo-proteinase 1 (TIMP 1) and enhancement of fiber assembly in Section 4.4.4, MMPs in Section 4.4.5, antioxidants in Section 4.4.6, heavy metal binding proteins and anti-inflammatory mediators in Section 4.4.7 and, finally, inflammatory factors in Section 4.4.8. Additionally, where known, the mechanisms of action that include antioxidant, reducing oxidative stress and anti-aging influences are presented throughout Section 4, which is the main portion of this review. Each main topic or section is self-contained with brief background information for each topic followed by examples/figures showing applications and/or analysis for the cited studies presented. Emphasis in this overview includes coverage from studies published from 2010 to 2016 (using the search terms resveratrol or equol and human skin and/or gene expression employing the Web of Science database, which includes the Web of Science core collection, Medline, Biosis Citation Index, Food Science and Technology Abstracts (FSTA)-the food science resource, KCI-Korean Journal Index, Russian Science Citation Index, the SciELO Citation Index from Latin America, Spain and South Africa and the Zoological Record) with earlier journal reports incorporated into the text where appropriate.

## 2. Chemical Structure Comparison of the Polyphenolic/Phytoestrogen Cosmeceuticals to 17β-Estradiol

In higher plants, thousands of molecules have polyphenolic structures that are thought to be involved in protecting plants against ultraviolet radiation, aggression by pathogens or stress-related responses such as drought or other extreme environmental conditions [26,27,28,29]; see [26] for a review of polyphenolic compounds. For example, wild Alaska berries exposed to harsh environmental conditions displayed high antioxidant and anti-inflammatory bioactivity of phenolic molecules compared to commercial berries [28].

**Figure 1:**
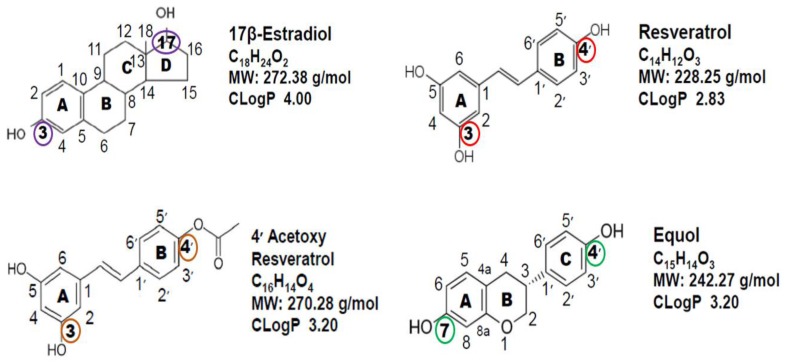
Comparison of the chemical structures, molecular formulas, molecular weights and CLogP values among 17β-estradiol, resveratrol, 4′ acetoxy resveratrol and equol. CLogP = the logP value of a compound representing its partition coefficient and lipophilicity. The purple circles at carbon 3 and 17 for 17β-estradiol represent the functional positions; the red and brown circles at carbon 3 and 4′ represent the functional positions for resveratrol and 4′ acetoxy resveratrol, respectively, and the green circles at carbon 7 and 4′ represent the functional positions for equol.

Polyphenolic molecules are defined by their chemical ring structures that have several hydroxyl groups on aromatic rings, which are classified as phytoestrogens due to their: (a) similar chemical structures to the most potent sex steroid hormone, 17β-estradiol; (b) ability to bind to the ER system; and (c) comparable molecular weights to 17β-estradiol (17βE2) [14,19,20,26] (Figure 1).

The two-dimensional representation of the chemical structures and formulas of 17βE2, resveratrol, the analog of resveratrol, 4′ acetoxy resveratrol, and equol are displayed in Figure 1. It should be pointed out that while endogenous estrogen hormones such as 17βE2 are steroids with a cyclo-hexane-phenanthrene parent chemical structure (i.e., the A, B and C ring) derived from cholesterol, the polyphenolic molecules shown in Figure 1 are not steroids, but are derived from plant sources.

However, the confirmation of the ring structures of the polyphenolic molecules resembles that of 17βE2. Furthermore, the lipophilic index (i.e., CLogP values) is somewhat similar among the polyphenolic molecules, which is slightly below that of 17βE2, suggesting similar penetration into cellular membranes and tissues with a lipophilic content like skin. Notably, 17βE2 has two functional hydroxy groups at carbon numbers 3 and 17 that enable it to bind to the ER system, and remarkably, the polyphenolic molecules have two functional hydroxyl groups in similar positions to that of 17βE2 at carbon numbers 3 and 4′ for resveratrol and 4′ acetoxy resveratrol and carbon numbers 7 and 4′ for equol, which are thought to contribute to their phytoestrogenic properties [10,11,12,26,30]. However, another perspective suggests that ER binding is only part of the story for phytoestrogens as far as their biological effects [26,31].

## 3. ERα and ERβ Binding Characteristics of 17-βEstradiol Compared to Resveratrol, 4′ Acetoxy Resveratrol, Racemic Equol, *S*-Equol and *R*-Equol

Estrogen receptors (ERα and ERβ) are members of the superfamily family of nuclear hormone receptors [32]. The “classical” estrogen receptor (ERα) was discovered by Elwood Jensen in 1958 and cloned in 1986, while ERβ was reported 10 years later in 1996 [32]. Human ERβ is homologous to ERα, particularly in the DNA-binding domain (97% amino acid identity) and in the ligand-binding domain (54% amino acid identity), but shares little homology in other domains [32]. Based on the dissimilar amino acid identities in the ligand-binding region, one may predict that 17β-estradiol would display different affinities for the ERs, but surprisingly, 17β-estradiol has almost equal high affinity for ERα and ERβ (Table 1).

Since receptor binding studies may or may not use the exact same methodologies or a single study may not test all polyphenolic compounds in question, data from two studies are displayed in Table 1 for the estrogen receptor binding characteristics for the cosmeceuticals covered in this overview. One research group examined a variety of phytoestrogens where the binding affinities were calculated in transient transfection and transactivation assays [10]. For resveratrol, its IC_50_ for ERα was 7.7 µM, while ERβ was 29.0 µM, which suggested that resveratrol is a weak agonist for both ERs at more than 1000-times less than 17βE2. To date, the resveratrol analog, 4′ acetoxy resveratrol, has not been examined for the estrogen receptor binding. However, later reports suggested that resveratrol and its analogs have selective estrogen receptor modulatory (SERM) properties [33,34,35]. For racemic equol, its IC_50_ for ERα was 1.5 µM, while ERβ was 0.2 µM [10]. This suggested that racemic equol had greater affinity for ERβ versus ERα by almost eight-fold with overall estrogenic properties greater than that of resveratrol.

In another study, the group used hERα and rERβ expression vectors in saturation isotherms experiments to determine the ER binding affinities of 17βE2 along with *S*-equol and *R*-equol [36]. The dissociation constants for 17βE2 were almost identical between ERα and ERβ that displayed very high affinity for both the ERs (Table 1). *S*-equol binds ERβ with approximately 20% affinity as does 17βE2 while having low affinity for ERα [36]. Thus, *S*-equol is classified as an SERM with high affinity for ERβ. Conversely, *R*-equol has weak affinity (approximately 1% or less to that of 17βE2) for either ER subtypes and, in general, has weak estrogenic properties at best [36]. The results in Table 1, have been corroborated by other reports [37,38,39]. To date, the binding characteristics of ERβ variants have not been studied in human skin for either *S*-equol or *R*-equol.

Finally, evidence exists that selective ERβ agonistic actions provide protective mechanisms in breast cancer and prostate health, which may be suggested for dermal applications, as well. For example, under normal physiological conditions, ERα mediates the proliferative actions of 17βE2, which are opposed by ERβ [40]. For example, in human mammary epithelial cells, ERα is expressed at 10–20% while ERβ is expressed at 80–85%, and in breast cancer cells, the expression of ERα is increased while ERβ is decreased [41,42,43]. However, ERβ levels are linked to better prognosis, increased survival and better response to anti-estrogen therapy [6,7]. Thus, the ratio of ERα to ERβ is considered to be a better prognostic biomarker of diagnosis and therapy of breast cancer [43]. For prostate health, activating ERα can have negative effects like increased proliferation, inflammation and malignancy, whereas, activating ERβ may have anti-proliferative, anti-inflammatory and anti-carcinogenic influences [44]. These data emphasize the importance and complexity of estrogen hormone action within the breast and prostate and highlight the known capacity of estrogens to exert both beneficial and adverse effects via ERβ and ERα, respectively. Thus, since there is high ERβ expression in human skin, this theme of protective mechanisms via this ER subtype may have applications to this important organ, which is covered in detail (i.e., ERβ activation, oxidative stress, inflammation, photoprotection, dermal thickness, etc.) elsewhere [7,14,45,46,47].

## 4. Human Skin Gene Expression

### 4.1. Gene Array (General)

Human skin was among the first organs to be studied by gene arrays due to its important barrier/defense function. Furthermore, commercial companies use gene arrays for testing and validation since the stratum corneum may be regulated and repaired by over-the-counter products containing cosmeceutical ingredients to address natural (chronological) aging along with superimposed photoaging [2,3,4,5,14,19,20,48,49,50].

### 4.2. Cosmeceuticals

The term cosmeceuticals coined by Kilgman in 1984 raised the bar for skin ingredients to include: (a) the ability of the active ingredient to penetrate the stratum corneum at an effective concentration; (b) specific beneficial mechanism(s) of action in the skin; and (c) published peer-reviewed studies/journal articles on the basic and clinical aspects of the active ingredient(s) that support its efficacy [51]. Since that time, polyphenolic compounds as active ingredients in skin products with different applications have been reported that cover all three criteria proposed by Kilgman [52,53,54]. As for the first benchmark, resveratrol, 4′ acetoxy resveratrol, racemic equol, *R*-equol and *S*-equol have been shown to penetrate human skin at effective concentrations using standard solubilizing agents [19,27,55,56,57].

### 4.3. Phase II Metabolism in Skin

It is important to compare phase II metabolism of the liver to that of skin in humans. For example, as stated previously, human liver phase II metabolism is known to conjugate polyphenolic compounds at a high level, especially resveratrol, when taken orally [15].

It is known that phase II metabolism (conjugation) does occur in human skin [58]. In fact, the use of skin explants demonstrated full coverage for glucuronidation, sulfation, *N*-acetylation, catechol methylation and glutathione conjugation, and investigators and cosmetic companies have explored the applications of natural compound analogs to address this issue [57,58,59]. However, the conjugation enzymatic activities in human skin are in the pmol/mg skin/h range. As noted by Manevski et al. “comparison of the skin and liver organ clearances implies that the skin contributes only marginally to the total systemic phase II enzyme-mediated metabolic elimination in humans” [58].

This concept was tested, in part, in a recent study where the gene array results of natural resveratrol versus 4′ acetoxy resveratrol were compared after 24-h exposure [57]. If the 4′ acetoxy resveratrol analog was hydrolyzed or rapidly metabolized in human skin, it may have less stability and/or act as a pro-drug in its delivery, and the obtained results of known skin-related genes may be similar to that of natural resveratrol. This was not the case. In fact, the resveratrol analog directly activated the SIRT 1 gene by almost two-fold above that of natural resveratrol that confirmed, at least in part, that phase II metabolism in human skin does not significantly alter compound efficacy. Thus, from this report, it is reasonable to suggest that only a minor portion of the test compound undergoes phase II metabolism in human skin, remains intact and does not act as a pro-drug [57,58].

### 4.4. Skin Gene Expression among Resveratrol, 4′ Acetoxy Resveratrol, R-Equol, Racemic Equol or S-Equol

#### 4.4.1. Background-Gene Arrays of the Cosmeceuticals

In a series of peer-reviewed journal reports, the cosmeceutical compounds were tested at 1% with 24-h exposure, under similar in vitro conditions using MatTek epidermal full thickness (EFT) human skin cultures where qPCR methodology quantified the percentage of stimulation or inhibition of 28 biomarkers compared to controls [27,55,56,57]. In the text (below in each subsection), under each gene expression category, the biological mechanism of how each compound tested may function is provided; however, several of the biochemical/molecular actions are currently unknown.

#### 4.4.2. Anti-Aging Gene Expression

Sirtuin 1 Activator

As shown in Table 2, the anti-aging biomarker results are displayed. Sirtuin 1 activator (SIRT 1) has received much attention for its regulatory role, mainly in metabolism and aging by acting as a deacetylating agent of many molecular and protein targets in numerous cells and tissues including the skin [19,20,50,60,61]. 4′ acetoxy resveratrol displayed the highest SIRT 1 expression, almost two-fold above that of resveratrol, while equol was not tested for this biomarker. The results demonstrated that the addition of an ester group at the 4′ carbon of resveratrol significantly increased SIRT 1 gene stimulation (Table 2). However, in regards to equol, in a recent study, the combined administration of racemic equol with natural resveratrol enhanced SIRT 1 expression in endothelial cells, which suggested an unknown positive biological action of equol even though it does not directly activate SIRT 1 [62]. Finally, an interaction of SIRT 1 with matrix metalloproteinases (MMPs) has been reported [63,64] (see below, Section 4.4.5 for MMPs). Apparently, SIRT 1 has been shown to inhibit MMPs either directly or indirectly, which has important implications on enhancing collagens and elastin to promote dermal health [63,64]. For example, Lee et al. demonstrated that SIRT 1 negatively regulated the expression of MMP-9 in skin tissue, while Ohguchi et al., showed that SIRT 1 inhibits MMPs in human dermal fibroblasts [63,64].

Proliferating Cell Nuclear Factor

Proliferating cell nuclear factor (PCNA) is a biomarker involved in DNA repair, and it is known to decrease in aged skin [65,66]. For PCNA, resveratrol displayed the highest gene stimulation, followed by the 4′ analog of resveratrol at 70%, while equol and its isomers had levels at approximately 40% to 30% to that of natural resveratrol (Table 2). Since resveratrol and presumably the 4′ analog are greater ERα agonists compared to equol or its isomers, this may explain, to some extent, the higher stimulation of *PCNA* gene expression for an interaction of ERα with PCNA that has been reported [67]. However, another group reported that two estrogen response element sequences near the PCNA gene are not responsible for its estrogen-enhanced expression [68]. Therefore, while there is some evidence to support a hormonal action with PCNA, the estrogenic mechanism leading to increase PCNA expression remains to be elucidated.

Nerve Growth Factor

Nerve growth factor (NGF) is a biomarker involved in: (a) skin/tissue repair for wound healing by the stimulation of collagen; (b) cell survival from UV exposure; and (c) the regeneration of cutaneous nerves as a neurotrophic factor [69,70,71,72]. *R*-equol displayed the highest gene stimulation, followed by racemic equol at 85% and then *S*-equol at 48%, while resveratrol and 4′ acetoxy resveratrol showed approximately 20% stimulation to that of *R*-equol (Table 2). The obtained pattern or trend profile (equol to resveratrol) is opposite that of the PCNA results.

Estrogenic stimulation of NGF and other neurotrophins, as well as their interactions, especially during development, is well established [73]. However, it is not clear how the estrogenic stimulation occurs via the ER subtypes. One group reported that ERα mediated the effects of estrogens on NGF-induced neurite sprouting or on cell survival to oxidative stress [74]; while another group demonstrated the modulation of NGF in peripheral organs by estrogen (and progesterone), which presents a complex picture for NGF expression [75].

5α-Reductase Type I

The 5α-reductase type I enzyme, contained in dermal fibroblasts and hair follicles, converts testosterone to the more potent androgen, 5α-DHT, which has a negative impact on skin and scalp hair health [9,47,76,77,78]. Neither resveratrol nor 4′ acetoxy resveratrol had any influence on 5α-reductase type I gene expression (Table 2), whereas racemic equol showed significant inhibition, which suggested a protective role for skin repair and against skin aging for this biomarker since androgens are known to stimulate MMPs that impair wound healing and scalp hair growth [9,14,47,76].

Current pharmaceuticals include inhibitors of the 5α-reductase enzyme particularly for prostate health and retention/growth of scalp hair where the chemical structure of many compounds has a modified steroid ring confirmation [78]. While the mechanism of how racemic equol inhibits the 5α-reductase enzyme in human skin is unknown, it may be related to its polyphenolic chemical structure being similar to 17βE2 and its associated SERM properties [14,27]. Additionally, both equol isomers are known to specifically bind free 5α-DHT that have applications not only to skin, but also prostate health [79].

Calcium-Binding Proteins (S100) A8 and A9

Finally, the calcium-binding proteins (S100) A8 and A9 and their gene biomarkers are associated with skin aging, inflammation and photoaging [80,81]. Furthermore, cells that progress through the spinous layer accumulate increasing amounts of protein involved in normal terminal differentiation, and S100 is one of these binding proteins [82,83]. For both S100 A8 and A9 biomarkers, racemic equol displayed the highest stimulation, followed by *R*-equol at approximately 88%, then *S*-equol at 25%, resveratrol at 14% and 4′ acetoxy resveratrol at about 10% of racemic equol values (Table 2).

While it is known that S100 A8 and A9 levels increase with skin aging [80,81], there is also a link to this protein’s expression in psoriasis and in breast cancer, where strong expression and secretion of S100A8/A9 may be associated with the loss of ERα in breast cancer [84]. Another group examining neuro-cortical outcomes in rodents found that ERα agonists regulated S100A8/A9 gene expression, which suggested an estrogenic link [85]. However, the regulation of S100A8/A9 in human skin remains unclear as to the estrogenic mechanism of regulation via the ER subtypes. For example, in general, resveratrol may be a better ERα agonist, but displayed low gene inhibition compared to racemic equol or *R*-equol for the S100A8/A9 biomarkers.

In brief, the aging skin biomarkers with the highest gene expression are: 4′ acetoxy resveratrol for SIRT 1; resveratrol for PCNA; *R*-equol for NGF; racemic equol for 5α-reductase type I; and the calcium-binding proteins A8 and A9 (Table 2). Remarkably, *S*-equol among the other cosmeceuticals tested did not stimulate or inhibit the aging biomarkers to the greatest extent. Finally, the mechanism(s) of how the cosmeceuticals stimulate or inhibitor the biomarkers above remains, for the most part, unknown, but their similar chemical structures to that of 17βE2 may provide some insight since the inhibition of estrogen biosynthesis is a common factor among phytoestrogen compounds [26].

#### 4.4.3. Extracellular Matrix Proteins

Collagens

The collagens provide the structural framework, while elastin fibers possess the elastic “bounce-back or recoil” properties of the dermal layer, which play essential roles in maintaining good skin health [7,8,9,14,19,20]. It is well known that estrogen improves skin quality and dermal health, especially in postmenopausal women by increasing skin collagen and elastin deposition, as well as hydration [6,7,8,14,45,46,47].

Collagen type I showed the greatest gene expression with racemic equol by approximately two-fold, while resveratrol, followed by *R*-equol and then *S*-equol displayed comparable, but slightly lower levels of stimulation, whereas the 4′ resveratrol analog did not alter this biomarker in a significant manner (Table 3). Conversely, resveratrol and 4′ acetoxy resveratrol both significantly stimulated collagen type III and IV by 2.3- and 1.7-fold, respectively, whereas equol or its isomers did not. The finding for racemic equol not stimulating collagen type III gene expression is intriguing since in vitro studies showed stimulation of collagen type III by 3.2-fold from our laboratory, which was greater than that of resveratrol or 4′ acetoxy resveratrol at approximately two-fold [27]. The difference in these results may be accounted for in the in vitro studies that used eight-week organotypic fibroblast cultures and the slow turnover rate of collagen type III compared to the short-term gene array experimental conditions [27].

In another study, tamoxifen, an antagonist of ER subtypes, was used to determine the potential estrogenic mechanism by which racemic equol’s beneficial influences on skin health for collagen type I and collagen type III occurred. When racemic equol plus tamoxifen was incubated together, a significant 3.1-fold decrease in collagen type I and a 4.6-fold decrease in collagen type III was recorded compared with equol alone [27]. This observation provides evidence, in part, for racemic equol’s affinity for ER subtypes and the potential mechanism by which it acts as a positive chemical messenger in skin. Since there is an abundance of ERβ in human skin, presumably this is the ER subtype that mediated these effects [27].

Elastin

For elastin gene expression, 4′ acetoxy resveratrol displayed the highest stimulation at 2.8-fold, while resveratrol, *R*-equol and racemic equol showed comparable, but lower levels around 1.8- to 1.9-fold, while *S*-equol did not significantly alter gene expression of elastin at all (Table 3). These results are interesting since all of the cosmeceuticals stimulated elastin gene expression in a significant manner by approximately two- to 2.8-fold, while *S*-equol did not. The reason for the lack of elastin gene stimulation by *S*-equol is unknown. However, as a general observation, these results are impressive since the turnover rate for elastin is low, and the maximum value of elastin stimulation may be around a two- to almost three-fold increase, regardless of the active cosmetic ingredient tested [14,27,56,57,86].

Where data are available from another study, when racemic equol plus tamoxifen was incubated together, a significant 2.7-fold decrease in elastin was recorded in in vitro organotypic eight-week skin cultures, which suggested an estrogenic link via ERβ for elastin regulation in human skin [27].

In summary, racemic equol displayed the highest stimulation for collagen I, while resveratrol and 4′ acetoxy resveratrol (4AR) shows the highest levels of collagen type III and IV, and 4AR had the most abundant stimulation of elastin followed by racemic equol, resveratrol, then *R*-equol. Notably, *S*-equol did not stimulate elastin at all.

#### 4.4.4. Inhibitor of MMPs (TIMP 1) and Enhancement of Fiber Assembly (LOX)

Tissue Inhibitor of Matrix Metalloprotease 1

Since oxidative stress directly or indirectly activates MMPs, the tissue inhibitors of matrix metalloproteases (TIMPs) are important skin enzymes that inhibit the actions of MMPs [14,87,88]. The TIMPs are known to decrease with exposure to UV light and with aging, which contributes to dermal matrix degradation, impaired cell growth and survival [88,89]. It is known that increased TIMP expression and activation blocks the negative influence of MMPs in causing skin damage [87,88,89].

For racemic equol, one study reported TIMP 1 gene stimulation at two-fold, while in another study, at 5.4-fold, stimulation was observed, whereas *S*-equol displayed a 1.5-fold stimulation and *R*-equol almost no activation of TIMP 1 expression (Table 4). Resveratrol was reported at 2.2-fold stimulation for TIMP 1, while 4′ acetoxy resveratrol displayed a 2.5-fold increase (Table 4). It is known that estrogen increases TIMP 1 and decreases MMPs in skin, but this theme is also seen in cultured human breast cancer cells, bone and heart tissue where estrogen’s positive effects can be reversed by tamoxifen, suggesting mediation via ERs [89,90,91,92,93,94]. To date, in human skin, the ER subtypes regulating TIMP 1 expression are unclear, but there is some evidence in other tissue sites that ERα may be involved [89,90,91,92].

Lysly Oxidase (LOX)

Lysly oxidase (LOX) and lysl oxidase-like (LOXL) enzymes are involved in cross-linking microfibrils (collagen) and/or elastin fibers [86,95,96]. The ultrastructural localization of LOXL was associated with elastin, while the interactions with collagen were presumably apparent [90]. LOX was found on collagen fibers associated with elastin. LOXL and LOX were detected in keratinocytes where LOX was mainly expressed by differentiating keratinocytes, in contrast to LOXL that was found in both proliferating and differentiating fibroblasts [95].

One group, over a period of time, reported that a dill extract induces LOXL gene expression [97,98]. Furthermore, like the other gene biomarkers above, there is evidence for estrogen regulation of LOX in other tissue sites and cells [99]. Only resveratrol and 4′ acetoxy resveratrol were tested for LOX. Natural resveratrol stimulated LOX gene expression by 1.8-fold, while the 4′ analog increased LOX to a similar level (by 1.9-fold) (Table 4).

#### 4.4.5. Matrix Metalloproteinases

Matrix metalloproteinases play an important role in the maintenance of the normal tissue architecture of human skin by connective tissue remodeling of the deposition, assembly and turnover of extracellular matrix macromolecules, such as collagen, elastin, etc. [100]. There are multiple MMPs that have descriptive names that will be initially introduced, but historically have been denoted by numerical designations that will be utilized herein [100]. While not providing exhaustive descriptions, the enzyme interstitial collagenase (MMP 1) has known substrates that can denature collagen types I, II, III, VII, VIII and X; stromeylsin-1 (MMP 3) has known substrates that can denature collagens I, IV, V, IX, X, XI and elastin; while gelatinase B (MMP 9) has substrates that can denature collagens IV, V, VII, X, XIV, elastin, etc. [100]. Stimulators of MMPs include: UV light, oxidative stress (free radicals), androgens, activator protein-1 (AP-1) and inflammatory factors/molecules, like interleukins, cytokines and pro-inflammatory transcription factor NF-κB (NF-κB) [9,14,27,47,100]. Inhibitors of MMPS include: estrogens, antioxidants and nuclear-factor-erythroid 2-related factor 2 (Nrf2), tissue inhibitors of matrix metalloproteinases (TIMPs), transforming growth factor β (TGF β) and retinoids [9,14,27,100].

Only resveratrol and equol along with its isomers were tested for their effects on MMPs (Table 5). For MMP 1, the greatest gene inhibition was seen with *R*-equol, followed by racemic equol at 60%, *S*-equol at 37% and resveratrol at 20% of *R*-equol values. Resveratrol did not significantly alter MMP 3 levels, while again, *R*-equol displayed the greatest gene inhibition followed by racemic equol at 90% and *S*-equol at 37% of *R*-equol values (Table 5). Again, *R*-equol displayed the greatest gene inhibition of MMP 9 while resveratrol was the lowest at 35%, followed by *S*-equol at 52% and racemic equol at 76% of *R*-equol levels. In summary, *R*-equol demonstrated the greatest gene inhibition for MMP 1, MMP 3 and MMP 9 among the compounds tested (Table 5). This *R*-equol inhibitory theme of the MMP genes was somewhat unexpected since it did not significantly stimulate TIMP 1 gene expression. However, it is known that equol and presumably its isomers are potent antioxidants, and evidence exists that it is a stimulator of Nrf2, which plays a key role in the cellular defense against oxidative stress by Nrf2’s capacity to induce the expression of numerous genes, which encode detoxifying enzymes and antioxidant proteins that provide protection in endothelia cells, skin morphogenesis, wound repair and skin cancer [101,102,103,104]. Finally, even though 4′ acetoxy resveratrol was not assayed for the MMPs, it likely possesses MMPs’ gene inhibitor characteristics similar to that of resveratrol.

#### 4.4.6. Antioxidants

There are some endogenous sources of antioxidants like glutathione and uric acid that are present in human skin [105,106]. However, many different antioxidants are obtained from dietary or exogenous sources like vitamin A, C and E and plant/food products that contain polyphenolic compounds, like resveratrol and equol, that are potent antioxidants [11,14,27,28,56,105,106].

In brief, the body has two major defense systems that include free radical detoxifying enzymes and antioxidant molecules [105]. The antioxidant capacity of the skin would be expected to be greater than that of internal organs due to its protective structure and the biological function of the dermal layers where the epidermis has the highest levels of antioxidant molecules and enzymes [106]. Catalase (CAT), superoxidase dismutase (SOD) and thioredoxin reductase (TXNRD) are antioxidant enzymes that protect against oxidative stress and the formation of oxygen free radicals [105,106].

Catalase (CAT), Superoxidase Dismutase (SOD) 1, SOD 2 and Thioredoxin Reductase (TXNRD)

For CAT and SOD 1, only resveratrol and 4′ acetoxy resveratrol were assayed. Resveratrol displayed the highest level of gene stimulation for CAT, while 4′ acetoxy resveratrol’s stimulation was 89% of that of resveratrol; and for SOD 1, resveratrol and its 4′ analog displayed identical values at a 1.6-fold stimulation (Table 6). For SOD 2, racemic equol showed the highest gene expression followed by 4′ acetoxy resveratrol at 85% and resveratrol at 80% of the racemic equol value. *R*-equol and *S*-equol were not assayed for SOD 2. Finally, only racemic equol stimulated TXNRD gene expression, while resveratrol and 4′ acetoxy resveratrol did not significantly increase TXNRD levels (Table 6). Thus, botanicals like resveratrol and equol appear to have properties with antioxidant activities beneficial for the enhancement of skin health [14,19,20,27,55,56].

#### 4.4.7. Heavy Metal Binding Proteins and Anti-Inflammatory Mediators

In general, like its cysteine-rich counterpart glutathione, metallothionein has the capacity to inhibit free radicals and to bind to toxic metals to enhance cellular detoxification [107,108]. Specifically, metallothionein (MTH) 1 and 2 can bind a wide range of metals including cadmium, zinc, mercury, copper, arsenic, silver, etc., and they can also serve as anti-inflammatory mediators in the body [107,108].

All of the cosmeceuticals tested displayed very high stimulation of MTH 1 gene expression (Table 7); however, the 4′ acetoxy resveratrol analog showed the greatest stimulation. For MTH 2, racemic equol displayed the greatest stimulation followed by 4′ acetoxy resveratrol at 63% and resveratrol at 39% of the racemic value, while *R*-equol and *S*-equol were not assayed (Table 7). Accordingly, the obtained results suggest that all of the cosmeceuticals tested provided high levels of metal detoxification capacity, protection against oxidants and, potentially, serve as mediators of inflammation [108].

#### 4.4.8. Inflammatory Factors

Research during the last two decades has revealed the mechanism by which continued oxidative stress leads to chronic inflammation, which in turn mediates most chronic diseases, including cancer and skin damage [109,110,111]. In this regard, it is well documented that many pro-inflammatory markers in human skin are increased with UV exposure [14,108,110]. Research on resveratrol and equol from plants and other sources has expanded the horizon for potential therapeutic remedies and treatments [4,11,14,19,20,27].

Interleukins

In general, for the interleukins (IL-1A, IL-1R2, IL-6 and IL-8), all of the cosmeceuticals tested displayed dramatically high inhibitory effects on the gene expression for these biomarkers (Table 8). In brief, resveratrol showed the greatest inhibition for IL-1A and IL-8, while racemic equol displayed the greatest inhibition for IL-1R2, and the 4′ resveratrol analog possessed the greatest inhibition for IL-6. In ranking the highest gene inhibitory impact to the lowest for the interleukins: resveratrol > *R*-equol > 4′ acetoxy resveratrol > racemic equol > *S*-equol (Table 8). The most remarkably result was that *S*-equol scored the lowest among the cosmeceuticals tested when it presumably possessed the highest affinity for ERβ in keratinocytes and dermal fibroblasts to enhance skin health [27,36]. The mechanism by which the cosmeceuticals inhibit gene expression of the interleukins is unknown.

Tumor Necrosis Factor

Tumor necrosis factor (TNF) is an inflammatory factor that can activate NF-κB, which can increase the inflammatory response to generate reactive oxygen species (ROS) along with a cascade of interconnecting events that lead to skin damage [14,110,111,112]. For the tumor necrosis factor super family 1 A (TNRSF 1A) gene, *R*-equol showed the greatest inhibition, followed by racemic equol at 80%, *S*-equol at 66%, resveratrol at 52% and then 4′ acetoxy resveratrol at 45% of the *R*-equol value (Table 8).

Cyclo-oxygenase 1 and 2

For the inflammatory factors, cyclo-oxygenase 1 and 2 (COX 1, COX 2), which are involved in prostaglandin production by either constitutive or inducible activities, respectively, both have been identified in human skin [113]. In fact, COX 2 has been shown to increase expression with human skin cancer [114]. In the present study, *R*-equol displayed the greatest inhibition for COX 1, followed by racemic equol at 74% and *S*-equol at 56% of the *R*-equol value (Table 8). Only resveratrol and its 4′ analog were tested for COX 2, where 4′ acetoxy resveratrol inhibition was 1.7-fold versus controls.

Therefore, for all of the skin-related gene biomarkers compared among the cosmeceuticals tested, all of the botanicals and subsequently improved compounds via laboratory methods potentially have utility as active ingredients in topical applications to enhance and/or improve dermal health.

## 5. Summary and Conclusions

Phytochemicals are botanical compounds and many are classified as phytoestrogens that are used in dermatology applications as cosmeceuticals. Resveratrol and equol are two of the best-known polyphenolic/phytoestrogens, where increased research attention has been directed to understand their properties and benefits for a variety of disorders and conditions, including dermal health. Estrogens are known to have protective and favorable influences on skin health, especially with aging and after menopause, whereas androgens oppose the positive actions of estrogens. Resveratrol and equol have similar chemical structures to 17β-estradiol with estrogen receptor (ER) binding characteristics that favor affinity for the ER subtypes. Human skin has an abundance of ERβ through which resveratrol and equol may act; however, some evidence suggests other mechanisms of action beyond the ERs for these cosmeceuticals. Phase II metabolism occurs in human skin, but at very low levels, implicating the stability of the applied cosmeceutical treatment.

Human skin gene expression is reported in this overview for 28 different biomarkers when resveratrol, 4′ acetoxy resveratrol, *R*-equol, racemic equol or *S*-equol was examined. A brief summary of all of the human skin biomarkers tested is outlined below by category to provide a quick analysis of the results. In the text (above), under each gene expression category, the biological mechanism of how each compound tested may function is provided. For example, equol may stimulate collagen/ elastin and TIMP 1 and inhibit MMPs via estrogenic mechanisms, whereas equol is known to activate the master gene Nrf2 for the production of a variety of antioxidants [14], which may or may not overlap with resveratrol or its 4′ analog. However, several of the biochemical/molecular actions for the gene array results are currently unknown.

The aging skin biomarkers have the highest gene expression: 4′ acetoxy resveratrol for SIRT 1; resveratrol for PCNA; *R*-equol for NGF; racemic equol for 5α-reductase type I; and thecalcium-binding proteins A8 and A9 (Table 2). Remarkably, *S*-equol among the other cosmeceuticals tested did not stimulate nor inhibit the aging biomarkers to the greatest extent.

For the extracellular matrix proteins, racemic equol displayed the highest stimulation for collagen I, while resveratrol and 4′ acetoxy resveratrol showed the highest levels of collagen type III and IV, and 4′ acetoxy resveratrol had the most abundant stimulation of elastin followed by racemic equol, resveratrol, then *R*-equol. Notably, *S*-equol did not stimulate elastin at all (Table 3). For racemic equol, one study reported TIMP 1 gene stimulation at two-fold, while in another study, 5.4-fold stimulation was observed, whereas *S*-equol displayed a 1.5-fold stimulation, and *R*-equol showed almost no activation of TIMP 1 expression (Table 4). Resveratrol was reported at 2.2-fold stimulation for TIMP 1, while 4′ acetoxy resveratrol displayed a 2.5-fold increase. Only resveratrol and 4′ acetoxy resveratrol were tested for LOX, where both stimulated gene expression by approximately 1.8-fold.

For the matrix metalloproteinases (MMPs), *R*-equol displayed the greatest inhibition of gene expression for MMP 1, MMP 3 and MMP9, followed by racemic equol, *S*-equol, then resveratrol (4′ acetoxy resveratrol was not tested for these biomarkers) (Table 5).

For the antioxidant biomarkers, only resveratrol and 4′ acetoxy resveratrol were tested for CAT and SOD 1, where both stimulated the gene expression from 1.6- to 1.8-fold. For SOD 2 and thioredoxin 1 (TXNRD 1), racemic equol displayed the greatest stimulation (Table 6).

For metallothionein gene expression, 4′ acetoxy resveratrol displayed the greatest stimulation for MTH 1 followed by resveratrol, *S*-equol, then racemic equol and finally *R*-equol, ranging from 64- to 21-fold. For MTH 2, racemic equol stimulated the gene expression by 5.1-fold followed by 4′ acetoxy resveratrol at 3.4-fold, then resveratrol at two-fold (Table 7).

For the inflammatory biomarkers: interleukin-1A showed the greatest inhibition with resveratrol followed by racemic equol, then *R*-equol (ranging from −22- to −13.9-fold); for interleukin-1 receptor II, racemic equol displayed the greatest inhibition followed by *R*-equol and, then, *S*-equol (ranging from −22.5- to −16.8-fold); 4′ acetoxy resveratrol showed the greatest inhibition for IL-6, followed by resveratrol, then *R*-equol (ranging from −35.2- to −5.5-fold); while resveratrol showed the greatest inhibition of IL-8, followed by *S*-equol, 4′ acetoxy resveratrol and then racemic equol (ranging from −7.9- to −3.5-fold); *R*-equol displayed the greatest inhibition of tumor necrosis factor at −3.1-fold and cyclooxygenase 1 (COX 1) at −3.6-fold; and finally, 4′acetoxy resveratrol inhibited COX 2 by −1.7-fold (equol or its isomers were not tested for COX 2) (Table 8).

Several studies and scientific commentaries over the last decade have pointed out that, in general, gene expression does not correlate with protein expression [115,116]. The fact that genome-wide correlation between expression levels of mRNA and protein hover around 40 percent is due to a variety of factors and conditions [115,116]. To address the criticism that the obtained gene expression results (via gene arrays) may not reflect the level of the gene product or changes in the protein expression of a given biomarker, our laboratory has studied racemic equol and reported the stimulation of collagen type I and elastin, and the inhibition of MMP 1 and MMP 3 gene expression values closely corresponded to the protein expression levels for these parameters [117]. Consequently, for these four gene biomarkers, there is evidence to suggest that the changes in gene expression via array analysis comprise a valid methodology to infer corresponding changes in protein expression levels. Thus, verification of mRNA levels can be used as a surrogate for corresponding protein levels, when both are tested [117].

In the present study, while all of the biomarkers were not validated via the quantification of changes in gene and protein expression levels, all investigators state that gene data are useful for identifying potential candidate biomarkers that may have promise in topical applications to improve dermal health especially when extra cellular matrix proteins like collagens, elastin and TIMPs are stimulated, and at the same time, the MMPs and inflammatory biomarkers are inhibited by an active skin ingredient like equol.

While it is difficult to rank the test materials for efficacy among the biomarkers, *S*-equol displayed promise as a topical, but in general, showed the lowest level of effectiveness compared to the other cosmeceuticals. The 4′ acetoxy resveratrol analog was more effective compared to resveratrol by approximately 1.6-fold. *R*-equol and racemic equol were almost equal in potency displaying greater inhibition vs. resveratrol or its 4′ analog, for the matrix metalloproteinases (MMPs), but among the inflammatory biomarkers, resveratrol, 4′ acetoxy resveratrol, *R*-equol and racemic equol displayed the highest inhibition.

Finally, although cosmeceutical research has advanced as far as the mechanisms of action via molecular and biochemical pathways and cascades along with efficacy, further study is warranted to understand the complexity of how polyphenolic and phytoestrogens compounds enhance and protect dermal health.

## Figures and Tables

**Table 1:** Estrogen receptor (ERα and ERβ) binding characteristics of 17β-estradiol compared to resveratrol, 4′ acetoxy resveratrol, racemic equol, *S*-equol and *R*-equol. 17β-estradiol has very high and almost equal affinity for ERα and ERβ regardless of the experimental methodologies employed. Resveratrol is considered a weak mix agonist for ERα and ERβ [10]. For [10], diethylstilbestrol (DES) was the standard. Equol has a chiral carbon and can exist as two isomers, *S*-equol and *R*-equol. As shown, *S*-equol binds ERβ approximately 1/5 as well as 17β-estradiol while having low affinity for ERα [36]. Thus, *S*-equol is classified as an SERM with high affinity for ERβ. Conversely, *R*-equol has weak affinity for either ER and, in general, has weak estrogenic properties at best [36]. However, the binding characteristics of ERβ variants have not been studied to date in human skin for either *S*-equol or *R*-equol. Noteworthy, *K*_d_ is the concentration of ligand needed to occupy 50% of receptors; *K*_i_ is reflective of the binding affinity; and the IC_50_ is more reflective of the functional strength of the inhibitor for the test material. Since the *K*_i_ takes into account the IC_50_ in its calculation, the *K*_i_ is reported more often.

Test Compound	Estrogen Receptor α	Estrogen Receptor β	Reference
17β-Estradiol	IC_50_ = 4.3 nM	IC_50_ = 5.7 nM	[10]
Resveratrol	IC_50_ = 7.7 µM	IC_50_ = 29.0 µM	[10]
4′ Acetoxy Resveratrol	Has not been studied, to date, for ER binding		
Racemic Equol	IC_50_ = 1.5 µM	IC_50_ = 0.2 µM	[10]
17β-Estradiol	*K*_d_ = 0.13 nM	*K*_d_ = 0.15 nM	[36]
*S*-Equol	*K*_i_ = 6.4 nM	*K*_i_ = 0.7 nM	[36]
*R*-Equol	*K*_i_ = 27.4 nM	*K*_i_ = 15.4 nM	[36]

**Table 2:** Human skin anti-aging gene expression among resveratrol, 4′ acetoxy resveratrol, *R*-equol, racemic equol or *S*-equol as the percentage of the increase (+) or decrease (−) compared to controls. Resveratrol data from [56]; 4′ acetoxy resveratrol data from [57]; racemic equol and *R*-equol and *S*-equol data from [27,55]; ● = data from [57]. NA = not assayed; NSA = no significant alteration in the gene biomarker.

Gene	Gene Name	Function	Resveratrol	4′ Acetoxy Resveratrol	*R*-Equol	Racemic Equol	*S*-Equol
*SIRT 1*	Sirtuin 1	Anti-aging factor	+180	+335	NA	↑ with resveratrol ●	NA
*PCNA*	Proliferating cell nuclear factor	DNA repair	+780	+540	+235	+285 to +300	+325
*NGF*	Nerve growth factor	Skin/tissue repair and neurotrophic factor	+800	+672	+3350	+2860	+1620
*5α-reductase 1*	5α-reductase type 1	Converts T to DHT and inhibits dermal health	NSA	NSA	NA	−180	NA
*S100 A8*	Calcium-binding protein A8	Skin aging, inflammation and photoaging	−340	−270	−2050	−2200	−580
*S100 A9*	Calcium-binding protein A9	Skin aging, inflammation and photoaging	−290	−160	−1850	−2250	−525

Since, these experiments were run in our laboratory under similar conditions (1% of the test compound applied and then exposed to human skin cultures for 24 h), the largest percent stimulation or inhibition for: (a) resveratrol is shown in red; (b) 4′ acetoxy resveratrol is shown in gold; (c) *R*-equol is shown in blue and (c) racemic equol is shown in green for all Tables. Data shown in black for a given gene biomarker among the compounds tested indicate no significant difference for the largest percent stimulation or inhibition of the displayed quantified values; however, values may be higher or lower among all of the compounds tested, but the highest or lowest rating was not color-coded due to no significant difference in the values. Remarkably, *S*-equol did not reveal any values that displayed a significant stimulation (highest) or significant inhibition (lowest) level(s) among the test compounds for any of the skin biomarkers tested. Note: racemic equol having more than one value for a given biomarker indicates the results of two independent experiments.

**Table 3:** Human skin extracellular matrix proteins gene expression among resveratrol, 4′ acetoxy resveratrol, *R*-equol, racemic equol or *S*-equol as the percentage of the increase (+) compared to controls.

Gene	Gene Name	Function	Resveratrol	4′ Acetoxy Resveratrol	*R*-Equol	Racemic Equol	*S*-Equol
*COL I alpha 1*	Collagen type I α1	Dermal fiber- structural support (most abundant in skin)	+225	NSA	+210	+235	+185
*COL III alpha 1*	Collagen type III α1	Abundant in youth	+230	+220	NSA	NSA	NSA
*COL IV alpha 1*	Collagen type IV α1	Separates/supports basement membranes	+160	+170	NSA	NSA	NSA
*ELN*	Elastin	Fiber-elastic/bounce-back properties	+180	+280	+175	+175 to +190	+1

**Table 4:** Human skin TIMP and LOX gene expression among resveratrol, 4′ acetoxy resveratrol, *R*-equol, racemic equol or *S*-equol as the percentage of the increase (+) compared to controls.

Gene	Gene Name	Function	Resveratrol	4′ Acetoxy Resveratrol	*R*-Equol	Racemic Equol	*S*-Equol
*TIMP 1*	Tissue inhibitor of matrix metallo-proteinase 1	Enzyme that inhibits actions of MMPs	+215	+250	+2	+200 to +540	+150
*LOX*	Lysyl oxidase	Cross links collagen and elastin fibers	+180	+190	NA	NA	NA

**Table 5:** Human skin matrix metalloproteinases (MMP) gene expression among resveratrol, 4′ acetoxy resveratrol, *R*-equol, racemic equol or *S*-equol as the percentage of the decrease (−) compared to controls.

Gene	Gene Name	Function	Resveratrol	4′ Acetoxy Resveratrol	*R*-Equol	Racemic Equol	*S*-Equol
*MMP 1*	Matrix metallo-proteinase 1	Breaks down collagens I, II and III	−180	NA	−890	−540	−325
*MMP 3*	Matrix metallo-proteinase 3	Breaks down collagens and elastin	NSA	NA	−885	−800	−330
*MMP 9*	Matrix metallo-proteinase 9	Remodels extracellular matrix	−485	NA	−1375	−1010 to −1080	−710

**Table 6:** Human skin antioxidant gene expression among resveratrol, 4′ acetoxy resveratrol, *R*-equol, racemic equol or *S*-equol as the percentage of the increase (+) compared to controls.

Gene	Gene Name	Function	Resveratrol	4′ Acetoxy Resveratrol	*R*-Equol	Racemic Equol	*S*-Equol
*CAT*	Catalase	Antioxidant enzyme protects against oxidative stress and ROS formation	+180	+160	NA	NA	NA
*SOD 1*	Superoxidase dismutase	Antioxidant enzyme protects against oxidative stress and ROS formation	+160	+160	NA	NA	NA
*SOD 2*	Superoxidase dismutase	Same as SOD 1	+160	+170	NA	+200	NA
*TXNRD1*	Thioredoxin reductase 1	Same as SOD 1 and 2; and anti-apoptotic	NSA	NSA	NA	+215	NA

**Table 7:** Human skin metallothionein gene expression among resveratrol, 4′ acetoxy resveratrol, *R*-equol, racemic equol or *S*-equol as the percentage of the increase (+) or decrease (−) compared to controls.

Gene	Gene Name	Function	Resveratrol	4′ Acetoxy Resveratrol	*R*-Equol	Racemic Equol	*S*-Equol
*MTH 1*	Metallothionein 1	Heavy metal binding protein and anti-inflammatory mediator	+4100	+6400	+2100	+1800 to +2310	+3840
*MTH 2*	Metallothionein 2	Same as MTH 1	+200	+340	NA	+510	NA

**Table 8:** Human skin inflammatory factor gene expression among resveratrol, 4′ acetoxy resveratrol, *R*-equol, racemic equol or *S*-equol as the percentage of the increase (+) or decrease (−) compared to controls.

Gene	Gene Name	Function	Resveratrol	4′ Acetoxy Resveratrol	*R*-Equol	Racemic Equol	*S*-Equol
*IL-1A*	Interleukin-1A	Inflammatory factor	−2200	−1010	−1385	−1700	−990
*IL-1R2*	Interleukin-1 receptor II	Inflammatory factor	−590	−190	−1730	−2250	−1675
*IL-6*	Interleukin-6	Inflammatory factor	−3200	−3520	−550	−455	−375
*IL-8*	Interleukin-8	Inflammatory factor	−790	−380	−295	−345	−445
*TNFR SF 1A*	Tumor necrosis factor super family 1A	Inflammatory factor that can activate NF-κB	−160	−140	−310	−250	−205
*COX 1*	Cyclo-oxygenase 1 or prostaglandin- endoperoxide synthase 1 (PTGS1)	Inflammatory factor, prostaglandin production (constitutive)	NSA	NSA	−360	−265	−200
*COX 2*	Cyclo-oxygenase 2 or prostaglandin-endoperoxide synthase 2 (PTGS2)	Inflammatory factor (inducible)	NSA	−170	NA	NA	NA
